# Betulinic Acid Affects the Energy-Related Proteomic Profiling in Pancreatic Ductal Adenocarcinoma Cells

**DOI:** 10.3390/molecules26092482

**Published:** 2021-04-24

**Authors:** Ching-Feng Chiu, Hsin-Yi Chang, Chun-Yine Huang, Chen-Zou Mau, Tzu-Ting Kuo, Hsiu-Chuan Lee, Shih-Yi Huang

**Affiliations:** 1 Graduate Institute of Metabolism and Obesity Sciences, Taipei Medical University, Taipei 11031, Taiwan; chiucf@tmu.edu.tw (C.-F.C.); hsinyi.chang@tmu.edu.tw (H.-Y.C.); ma48108005@tmu.edu.tw (C.-Z.M.); 2 Nutrition Research Center, Taipei Medical University Hospital, Taipei 11031, Taiwan; 3 TMU Research Center of Cancer Translational Medicine, Taipei Medical University, Taipei 11031, Taiwan; 4 Graduate Institute of Cancer Biology and Drug Discovery, College of Medical Science and Technology, Taipei Medical University, Taipei 11031, Taiwan; 5 Master Program in Clinical Pharmacogenomics and Pharmacoproteomics, College of Pharmacy, Taipei Medical University, Taipei 11031, Taiwan; 6 School of Nutrition and Health Sciences, Taipei Medical University, Taipei 11031, Taiwan; ma07107009@tmu.edu.tw; 7 Ph.D. Program for Cancer Molecular Biology and Drug Discovery, College of Medical Science and Technology, Taipei Medical University and Academia Sinica, Taipei 11031, Taiwan; d621107003@tmu.edu.tw

**Keywords:** pancreatic cancer, betulinic acid, proteomics

## Abstract

Pancreatic ductal adenocarcinoma (PDAC) is an aggressive disease with a 5-year survival rate of <8%. Therefore, finding new treatment strategies against PDAC cells is an imperative issue. Betulinic acid (BA), a plant-derived natural compound, has shown great potential to combat cancer owing to its versatile physiological functions. In this study, we observed the impacts of BA on the cell viability and migratory ability of PDAC cell lines, and screened differentially expressed proteins (DEPs) by an LC-MS/MS-based proteomics analysis. Our results showed that BA significantly inhibited the viability and migratory ability of PDAC cells under a relatively low dosage without affecting normal pancreatic cells. Moreover, a functional analysis revealed that BA-induced downregulation of protein clusters that participate in mitochondrial complex 1 activity and oxidative phosphorylation, which was related to decreased expressions of RNA polymerase mitochondrial (POLRMT) and translational activator of cytochrome c oxidase (TACO1), suggesting that the influence on mitochondrial function explains the effect of BA on PDAC cell growth and migration. In addition, BA also dramatically increased Apolipoprotein A1 (APOA1) expression and decreased NLR family CARD domain-containing protein 4 (NLRC4) expression, which may be involved in the dampening of PDAC migration. Notably, altered expression patterns of APOA1 and NLRC4 indicated a favorable clinical prognosis of PDAC. Based on these findings, we identified potential proteins and pathways regulated by BA from a proteomics perspective, which provides a therapeutic window for PDAC.

## 1. Introduction

Pancreatic ductal adenocarcinoma (PDAC) is an aggressive disease and causes the highest mortality rate and the lowest survival rate in the world [1], as well as in Taiwan [2]. Pancreatic ductal adenocarcinoma was the seventh leading cause of cancer-related deaths in Taiwan in 2019, and the number of deaths has also been increasing year to year [2]. The prognosis of pancreatic cancer patients is extremely poor, with a 5-year survival rate of approximately 8% [3]. Thus, exploring new treatment strategies with greater drug sensitivities and safer dosages is imperative.

Betulinic acid (BA), a lupane-type pentacyclic triterpenoid, is a phytochemical widely distributed throughout the plant kingdom in the Rhamnaceae, Paeoniaceae, Myrtaceae, and Betulaceae families. BA is most abundant in the bark of white birch trees [4]. BA has been confirmed to demonstrate various physiological activities, such as anti-inflammatory ability [5], inhibiting capability of replication of the human immunodeficiency virus (HIV) [6], and enhancement of immune response [7]. BA also shows selective toxicity towards many different types of tumors without damaging normal cells. The mechanisms of BA against cancer include promoting an endogenous apoptotic response [8], decreasing cancer cell stemness and the epithelial-to-mesenchymal transition (EMT) [9], and inhibiting tumor tissue angiogenesis [10]. Studies have also shown that co-administration of the multi-target kinase inhibitor, sorafenib, and BA in PDAC cell lines can reduce the proliferation and colony-forming ability of PDAC cell lines without inducing apoptosis [11]; others have found that the chemotherapeutic drug gemcitabine, combined with BA, significantly increased apoptosis of PDAC cell lines compared to an intervention with gemcitabine alone [12], suggesting that BA may synergize antitumor drugs by enhancing the drug’s efficacy and reducing drug resistance.

Proteomic technologies including mass spectrometry (MS), reverse-phase protein arrays, and protein–protein interaction mapping have played crucial roles in drug development [13]. Profiling protein expressions through proteomics identifies novel tumor cell biomarkers, and provides information about mechanisms of cancer development as well as protein–drug interactions [14]. A previous proteomic investigation into BA’s effects on cervical cancer HeLa cells suggested that BA triggers the endoplasmic reticulum pathway and reactive oxygen species (ROS)-mediated mitochondrial pathway, thereby inducing apoptosis of HeLa cells [15]. However, the effects of BA on PDAC from a proteomics view remain to be explored. Thus, in this study, we conducted a proteomics analysis of BA-treated PDAC cell lines to identify tumor-associated proteins, and further investigated their potential roles in PDAC cells.

## 2. Results

### 2.1. Selective Cytotoxicity of BA against Pdac Cells

To examine the cytotoxicity of BA against PDAC cells, we treated Mia PaCa-2, SUIT-2, and hTERT-HPNE cells with serial concentrations of BA for 24 and 48 h. IC50 values of Mia PaCa-2, SUIT-2, and hTERT-HPNE cells were 1.783, 0.505, and 5.689 μM at 24 h, and were 2.908, 0.864, 7.601 μM at 48 h (Table 1, Figure S1). This indicates that BA can impede PDAC cell growth at relatively low concentrations without affecting normal cells, suggesting that BA exhibits selective cytotoxicity.

### 2.2. Effect of BA on Pdac Cell Migration

**Figure 1:**
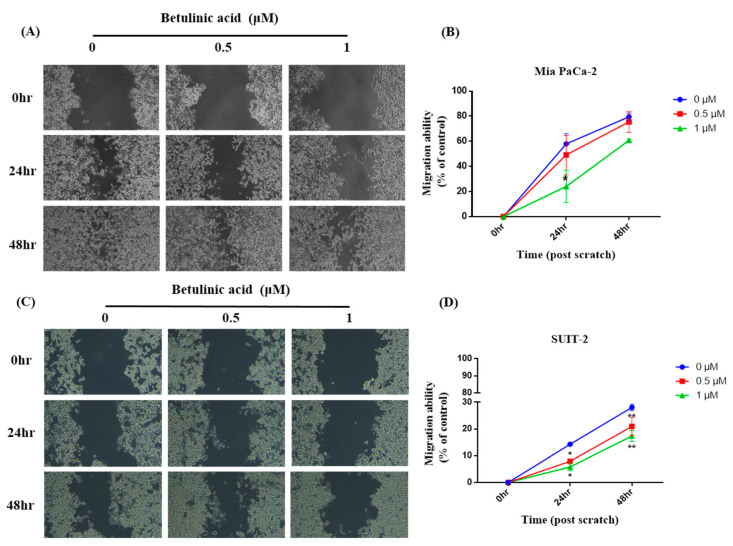
Effects of betulinic acid (BA) on Mia PaCa-2 and SUIT-2 cell migration. (**A**,**C**) Cells were treated with BA for up to 48 h at concentrations of 0, 0.5, and 1 µM. The cell migratory ability was evaluated by a wound-healing assay, and representative images were captured under a phase-contrast microscope at 20× magnification. (**B**,**D**) Quantitative analysis of the migration of Mia PaCa-2 and SUIT-2 cells was calculated using ImageJ software. The migratory ability is expressed as a percentage of the wound-closure area compared to that of the control (0 h). Data are expressed as the mean ± SEM (n = 2). * *p* < 0.05 and ** *p* < 0.01 compared to the 0 µM group at the same treatment period.

**Figure 2:**
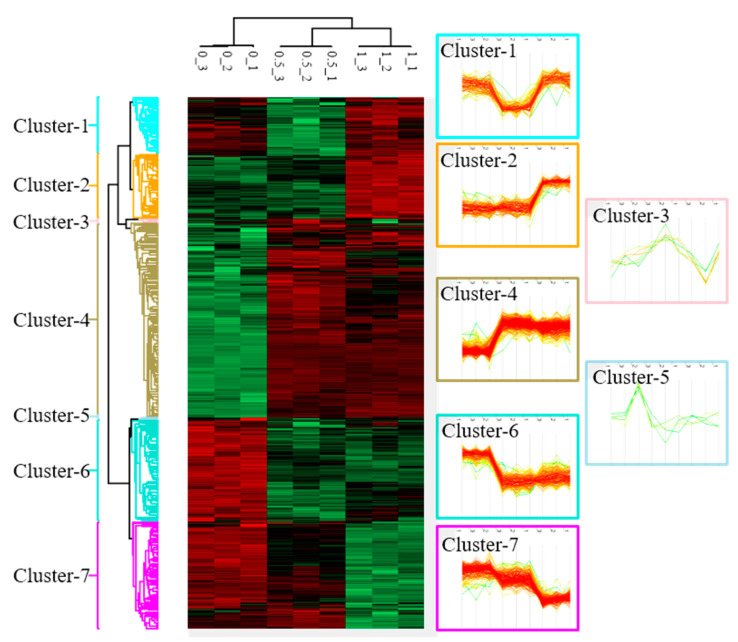
Cluster analysis of differentially expressed proteins (DEPs) in Mia PaCa-2 cells under different doses (0, 0.5, and 1 µM) of betulinic acid (BA) treatment. Samples of each concentration were analyzed in triplicate. After normalizing average values of logarithmic intensities of each protein profile, 3316 proteins were identified and subsequently classified into seven clusters with distinct expression patterns. An ANOVA was applied to determine the statistical significance of DEPs using the threshold of a false detection rate (FDR) of <0.05. Results are presented as a heat map.

To investigate if BA reduces the metastatic potential of PDAC cells, the migratory ability of primary tumor-derived Mia PaCa-2 cells and in liver-metastasized SUIT-2 cells was observed via a wound-healing assay. Our results showed that in Mia PaCa-2 cells, the wound-healing areas after 24 and 48 h in the 0.5 μM group showed no difference from those in the control group. However, the wound-healing area in the 1 μM group significantly decreased compared to the control group after 24 h; while there was a trend of a decline after 48 h. Furthermore, there was no difference between the 0.5 μM group and the 1 μM group (Figure 1). In SUIT-2 cells, the wound-healing areas in the 0.5 and 1 μM groups were also significantly lower than those in the control group after both 24 and 48 h, whereas no difference was observed between the 0.5 and 1 μM groups (Figure 2).

### 2.3. Identification and Clustering of BA-Regulated Deps in Mia Paca-2 Cells

To understand changes in protein profiles affected by BA, we used a protein microarray analysis to identify 3316 DEPs after an intervention with 0, 0.5, and 1 μM BA. According to changes in protein expression patterns responding to each dosage, these DEPs were classified into seven clusters (Figure 2). This study selected cluster 4, containing 260 significantly upregulated proteins (Table S1); cluster 6, containing 135 significantly downregulated proteins (Table S2); cluster 7, containing 141 dose-dependently downregulated proteins (Table S3) for subsequent gene function annotation.

**Figure 3:**
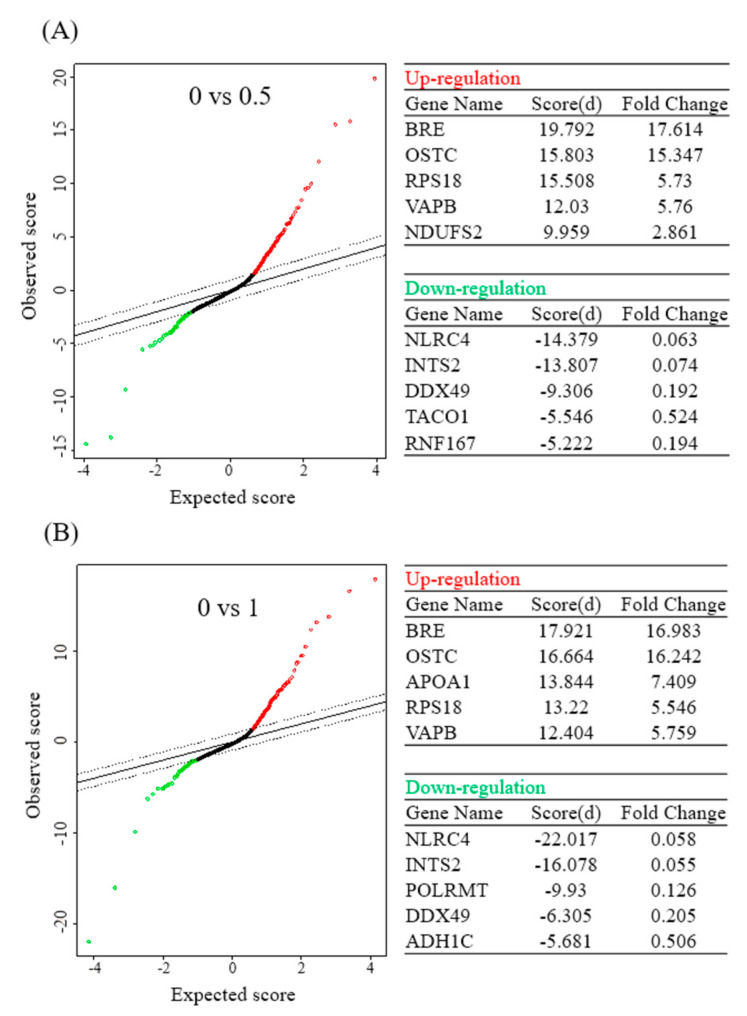
Significant analysis of microarray (SAM) in betulinic acid (BA)-treated Mia PaCa-2 cells and the corresponding control (no treatment). Tables show the top five differentially upregulated and downregulated protein-coding genes under 0.5 (**A**) and 1 µM (**B**) of BA treatment (with a false detection rate (FDR) of <0.01).

In addition, this study further analyzed results of the protein microarray with the SAM statistical tool, and obtained five upregulated proteins and five downregulated proteins with the highest multiples of change after the BA intervention at different concentrations. Upon treatment with 0.5 μM BA, protein expressions of BRE, OSTC, RPS18, VAPB, and NDUFS2 significantly increased, while those of NLRC4, INTS2, DDX49, TACO1, and RNF167 significantly decreased. Upon treatment with 1 μM BA, protein expressions of BRE, OSTC, APOA1, RPS18, and VAPB significantly increased (Figure 3A), whereas those of NLRC4, INTS2, POLRMT, DDX49, and ADH1C significantly decreased (Figure 3B).

### 2.4. GO and KEGG Analysis of DEP Clusters

Coding gene lists of clusters 4, 6, and 7 were input into the DAVID online analytical tool for functional annotation, with the GO and KEGG databases selected for analysis. Based on cluster 4, the GO analysis showed that the most significant enrichment of BP, CC, and MF was “rRNA processing”, “extracellular exosome”, and “poly (A) RNA binding”. As for the KEGG analysis, the most significant pathway enrichment was “ribosome” (Table S4). In cluster 6, the GO analysis indicated that the most significant enrichment of BP, CC, and MF was “mitochondrial electron transport, NADH to ubiquinone”, “mitochondrion”, and “NADH dehydrogenase (ubiquinone) activity”. Regarding the KEGG analysis, the most significant pathway enrichment was “metabolic pathways” (Table S5). In cluster 7, the GO analysis pointed out that the most significant enrichment of BP, CC, and MF was “cell–cell adhesion”, “extracellular exosome”, and “poly (A) RNA binding”. In terms of the KEGG analysis, the most significant pathway enrichment was “glycolysis/gluconeogenesis” (Table S6).

### 2.5. Oncomine and KM-Plot Analysis of BA-Induced Up- and Downregulated Proteins

Based on the SAM analytical results, the six most significantly upregulated and seven most downregulated proteins induced by BA were selected to be inputted into the Oncomine and KM-plot online databases.

**Figure 4:**
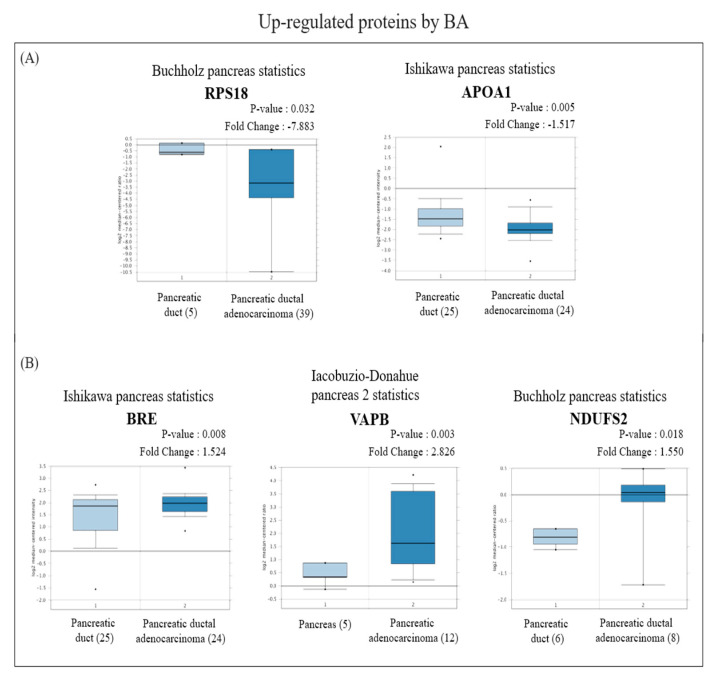
Analysis of the specific expressions of betulinic acid (BA)-induced upregulated proteins in clinically normal (left plot) and pancreatic ductal adenocarcinoma (PDAC; right plot) tissues using the Oncomine database. (**A**) Expressions of RPS18 (*p* = 0.032) and APOA1 (*p* = 0.005) were significantly lower in PDAC tissues compared to normal tissues. (**B**) Expressions of BRE (*p* = 0.008), VAPB (*p* = 0.003), and NDUFS2 (*p* = 0.018) were significantly higher in PDAC tissues compared to normal tissues.

**Figure 5:**
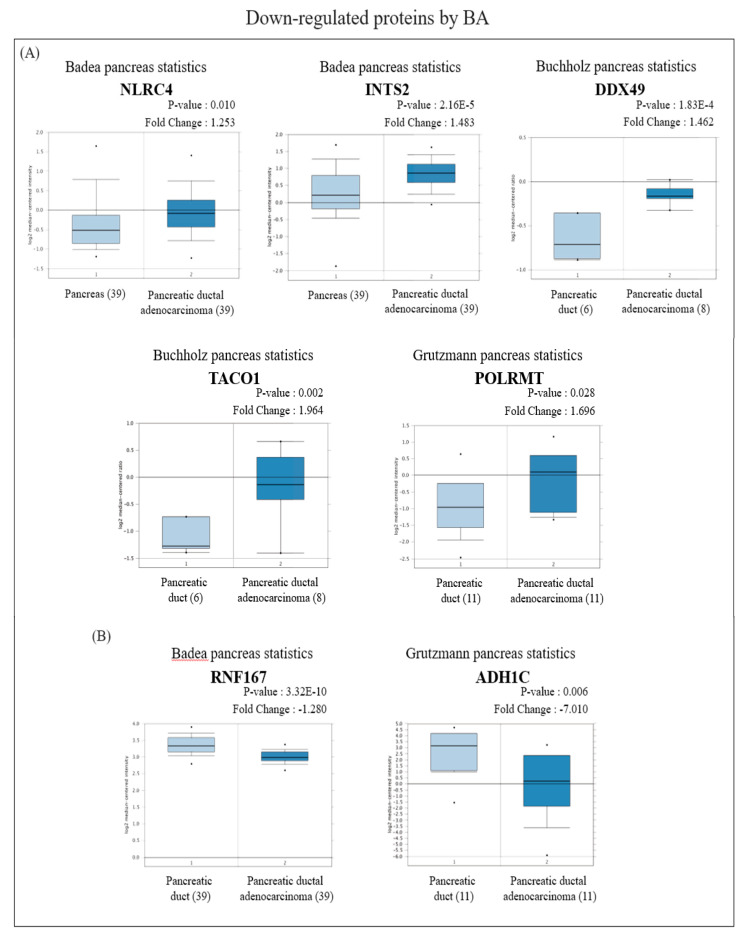
Analysis of the specific expressions of betulinic acid (BA)-induced downregulated proteins in clinically normal (left plot) and pancreatic ductal adenocarcinoma (PDAC; right plot) tissues using the Oncomine database. (**A**) Expressions of NLRC4 (*p* = 0.010), INTS2 (*p* < 0.001), DDX49 (*p* < 0.001), TACO1 (*p* = 0.002), and POLRMT (*p* = 0.028) were significantly higher in PDAC tissues compared to normal tissues. (**B**) Expressions of RNF167 (*p* < 0.001) and ADH1C (*p* = 0.006) were significantly lower in PDAC tissues compared to normal tissues.

In view of the Oncomine results, among the proteins with increased expression, messenger (m) RNA expressions of RPS18 and APOA1 in PDAC tissues were significantly lower than those in normal pancreatic tissues (Figure 4A), while BRE, VAPB, and NDUFS2 were significantly higher than those in normal pancreatic tissues (Figure 4B). On the other hand, among proteins with reduced expression, mRNA expressions of NLRC4, INTS2, DDX49, TACO1, and POLRMT in PDAC tissues were significantly higher than those in normal pancreatic tissues (Figure 5A), whereas RNF167 and ADH1C were significantly lower than those in normal pancreatic tissues (Figure 5B).

**Figure 6:**
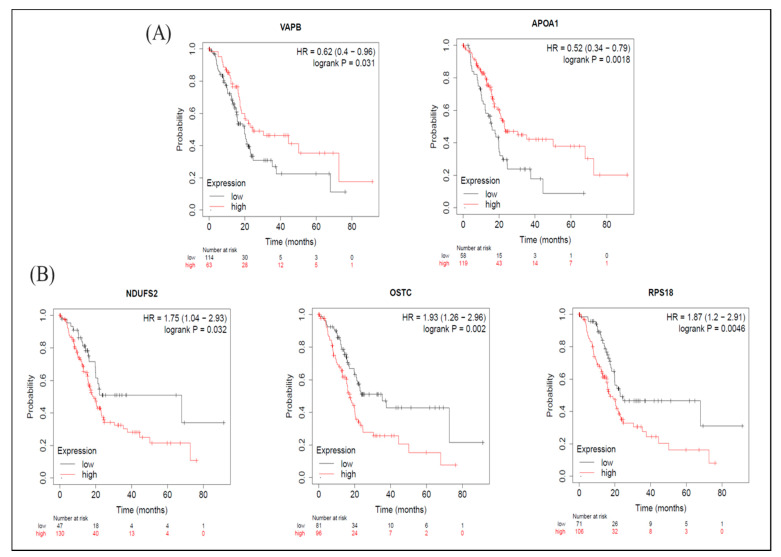
Meta-analysis of betulinic acid (BA)-induced upregulated proteins in overall survival (OS) in pancreatic ductal adenocarcinoma (PDAC) databases of the KM plotter. (**A**) High expressions of VAPB (*p* = 0.031) and APOA1 (*p* = 0.002) were associated with greater OS. (**B**) Contrarily, high expressions of NDUFS2 (*p* = 0.032), OSTC (*p* = 0.002), and RPS18 (*p* = 0.005) were associated with worse OS.

As for the KM-plot results, among proteins with increased expression levels induced by BA, in terms of OS, groups with higher expression levels of VAPB and APOA1 had longer survival periods (Figure 6A), while groups with higher expression levels of NDUFS2, OSTC, and RPS18 had shorter survival periods (Figure 6B). From the perspective of RFS, groups with higher expression levels of BRE (BABAM2) had longer survival periods (Table S7), whereas groups with higher expression levels of NDUFS2 and OSTC had shorter survival periods Table S7). The median survival times of high- and low-expression groups in upregulated proteins are shown in Table S7.

**Figure 7:**
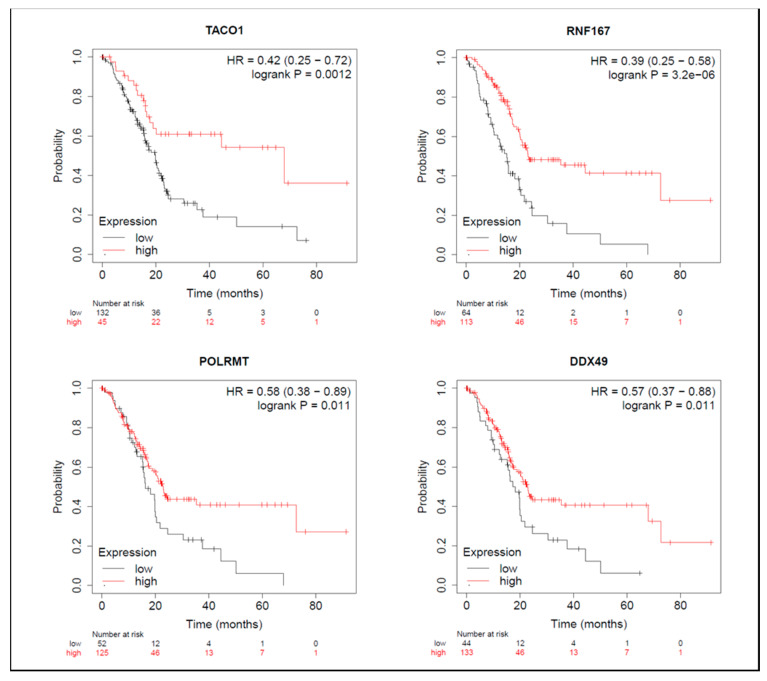
Meta-analysis of betulinic acid (BA)-induced downregulated proteins in overall survival (OS) in pancreatic ductal adenocarcinoma (PDAC) databases of the KM plotter. High expressions of TACO1 (*p* = 0.001), RNF167 (*p* < 0.001), POLRMT (*p* = 0.011), and DDX49 (*p* = 0.011) were associated with greater OS.

On the other hand, among proteins with reduced expression induced by BA, in terms of OS, groups with higher expressions of TACO1, RNF167, POLRMT, and DDX49 had longer survival periods (Figure 7). From the perspective of RFS, groups with higher NLRC4 expression had shorter survival periods (Table S8), while groups with higher expressions of RNF167 and POLRMT had longer survival periods (Table S8). Median survival times of high- and low-expression groups in downregulated proteins are shown in Table S8.

## 3. Discussion

In this study, the inhibited dosage of BA in Mia PaCa-2 and SUIT-2 cell growth was lower than that in hTERT-HPNE cells, this observation allows us to conclude that the applied dosage did not have cytotoxicity. Pisha E et al. [16] reported that BA inhibited the growth of melanomas in a mouse xenograft model, with long-term toxic-free effects observed under a high dosage of BA (500 mg/kg) treatment, suggesting that BA has a good therapeutic index. Additionally, Zuco V et al. also suggested that BA exhibits a significant inhibitory effect on cancer cell proliferation (IC50 of 1.5~4.5 μg/mL) including small-cell lung cancer, cervical cancer, and ovarian cancer; while in non-cancer cells such as blood lymphocytes, higher doses (IC50 of ≥10 μg/mL) were needed to show growth inhibition. In contrast, the clinical drug doxorubicin shows toxic effects on both cancer (0.014~0.34 μg/mL) and normal cells (0.02~0.38 μg/mL), with only slight differences in treatment dosages [17]. Hence, it is speculated that clinical PDAC patients may have better tolerance to BA than to other chemotherapeutic drugs.

The potential mechanism by which BA inhibits cancer cell proliferation is related to its apoptosis-inducing biological activity. BA promotes mitochondrial outer membrane permeabilization, which causes the release of cytochrome C and apoptosis-inducing factors into the cytoplasm, thus initiating the proapoptotic caspase-9/-3 cascade pathway [8]. In addition, loss of the mitochondrial membrane potential (MMP) induced by BA can also cause excessive accumulation of ROS in cancer cells and lead to cell death [18]. Evidence shows that cancer cells show functional abnormalities in mitochondria, including energy metabolism biased towards aerobic glycolysis, a rising transmembrane potential, and increased ROS production [19]. Therefore, we speculated that the BA-induced endogenous apoptotic response in mitochondria may partially explain its selective cytotoxicity towards cancer cells. In the present study, we found that downregulated proteins induced by BA were mainly related to mitochondrial complex I activity and the oxidative phosphorylation process of the respiratory chain, implying that BA may impede PDAC cell growth via altering mitochondrial function.

The migration and invasion processes are necessary for distant metastasis during PDAC progression [20]. PDAC was observed to exert high metastatic potential, which is frequently activated [21]. Recent studies found that BA can inhibit the EMT via increasing E-cadherin and decreasing vimentin expression, thereby inhibiting the migratory ability of PDAC [9]. Our results showed that after BA administration, the migratory ability of PDAC cells was significantly inhibited ad hoc in SUIT-2 cells, which echoes results of previous research.

It is noteworthy that dysfunction of mitochondria in cancer cells is also related to their metastatic potential. Studies showed that lung cancer cells with higher mitochondrial complex I activity and membrane potentials demonstrated greater migratory and invasive abilities, which could be decreased by complete inhibition of the electron transport chain [22]. Furthermore, increased ROS production during the process of dysregulated oxidative phosphorylation may promote the stable expression of hypoxia-inducible factor-1 α, subsequently causing occurrence of the EMT [23]. Meanwhile, ROS can also enhance the migratory plasticity of cancer cells by activating two tyrosine kinases, Src and Pyk2 [24]. In the present study, we assumed that altered mitochondrial complex I activity and oxidative phosphorylation under BA treatment may be partially responsible for the anti-migratory effect of BA. Importantly, we assigned the hub of up- and downregulated DEPs screened by the SAM analysis of proteomic data into mitochondrial function-related proteins (NDUFS2, POLRMT, and TACO1) and inflammation-related proteins (APOA1 and NLRC4).

NADH: ubiquinone oxidoreductase core subunit S2 (NDUFS2) is one of the main regulators of mitochondrial complex I that assists electron transfer to produce a proton gradient and facilitate subsequent ATP synthesis [25]. However, recent studies discovered a role of NDUFS2 in cancer. S100 calcium-binding protein A4, a protein related to cancer metastasis, can promote mitochondrial activity and ATP production via upregulating NDUFS2 expression, which is beneficial to the proliferation and invasion of pancreatic cancer SUIT-2 cells [26]. Our study found that NDUFS2 is highly expressed in PDAC tissues compared to normal tissues, and PDAC cohorts with high NDUFS2 expression are linked to worse prognoses, implying that NDUFS2 may have a negative impact. However, we also observed significant upregulation of NDUFS2 after BA treatment. The underlying mechanism has yet to be explained, but we believe that BA still possesses an effect of inhibiting mitochondrial function due to annotations of the downregulated protein cluster.

RNA polymerase mitochondrial (POLRMT) is an RNA polymerase that plays a key role in the initiation stage of the transcription of the mitochondrial genome [27]. Of note, it encodes transcription of 13 subunits in the enzyme complex related to oxidative phosphorylation [28]. A high degree of oxidative phosphorylation can often be observed in cancer models, so we speculated that POLRMT may be associated with cancer progression. So far, it was proven to be overexpressed in hematological malignancies, and knockdown of POLRMT expression significantly inhibited the growth of leukemia cells, mitochondrial complex I activity, and oxidative phosphorylation without affecting the cell cycle [29]. Additionally, overexpression of POLRMT increased the metabolic rate of breast cancer cells and promoted tumor growth in vivo, showing its tumorigenic properties [30]. In this study, we observed that PDAC tissues had higher POLRMT expression than normal tissues, and treatment with BA significantly inhibited POLRMT expression in Mia PaCa-2 cells. Therefore, we assumed that altered mitochondrial complex I activity may be related to the downregulation of POLRMT.

A translational activator of cytochrome c oxidase (CCO 1 or TACO1), namely mitochondrial complex IV, is known to catalyze the conversion of oxygen into water molecules for terminal electron transfer of the electron transport chain (ETC). Within this complex, TACO1 is the translational activation protein of CCO subunit 1 (COX1). Research showed that a missense mutation of TACO1 may cause reduced COX1 expression, accompanied by a CCO deficiency and an imbalance of oxidative phosphorylation, thereby leading to late-onset mitochondrial dysfunction [31]. Nonetheless, whether TACO1 plays a specific role in cancer still remains elusive. A database analysis in our study revealed that TACO1 had higher expression in PDAC tissues than in normal tissues, and BA treatment significantly inhibited its protein expression in vitro. In view of these findings, we speculated that BA could regulate highly activated oxidative phosphorylation in PDAC cells.

As shown by the KM-plot analysis, however, clinical PDAC patients with higher POLRMT and TACO1 expression levels tended to have an optimal prognosis; we postulated that these two proteins also play an indispensable role in maintaining normal mitochondrial energy metabolism. Therefore, treatments targeting POLRMT and TACO1 need to be evaluated in relation to whether they have a selective inhibitory effect on cancer cells, including BA in this study.

Apolipoprotein A1 (APOA1) is an apolipoprotein that constitutes one of the high-density lipoprotein (HDL) components, that acts as a cofactor to activate lecithin-cholesterol acyltransferase (LCAT) and as a ligand to interact with scavenger receptor class B type 1 (SRB1) in the liver, thus promoting reverse cholesterol transport (RCT) [32]. In addition, APOA1 also increases the secretion of nitric oxide (NO) and prostacyclin (PGI2) from vascular endothelial cells to promote vasodilation [33], thereby manifesting the cardiovascular protective effect of APOA1. Notably, APOA1 also has physiological effects in cancer. For instance, it can inhibit the aggregation of proinflammatory cells in the pancreatic cancer tumor microenvironment (TME), including Th1 and Th17 helper T cells [34], consequently exerting anti-inflammatory properties. In addition, APOA1 is capable of decreasing MMP-9 expression to reduce the invasive ability of cancer cells, and of inhibiting vascular endothelial growth factor (VEGF)-induced and basic fibroblast growth factor (bFGF)-induced vascular endothelial cell proliferation, migration, and tube formation, together preventing tumor angiogenesis [35]. Apart from these multiple physiological functions, studies indicated that serum levels of APOA1 in clinical PDAC patients are nearly two-fold lower than those of normal participants [36]. Our database analytical results showed that APOA1 expression in PDAC tissues was lower than that in normal tissues, and PDAC patients bearing higher APOA1 expression had better prognoses; moreover, APOA1 expression was also increased with BA treatment. Based on these findings, we assumed that APOA1 may partially explain the anticancer effect of BA.

NLR family CARD domain-containing protein 4 (NLRC4) is a member of the nuclear-binding oligomerization domain-like receptor (NLR) family. It can activate and assemble a multiprotein oligomer called the inflammasome, thereby initiating caspase-1-induced apoptosis, promoting maturation and secretion of interleukin (IL)-1 β and IL-18 to activate extracellular immune cells, and ultimately causing inflammation [37]. Of note, obesity is also regarded as chronic low-grade inflammation, with evidence of macrophage infiltration in adipose tissues. This obesity-induced endogenous immune response promotes activation of inflammasomes [38], and was found to be related to cancer progression. In tumor tissues of obese breast cancer patients, significant accumulation of tumor-associated macrophages accompanied by activated NLRC4 inflammasomes was observed, thus activating IL-1 β to promote VEGF-α (VEGFA) secretion by adjacent adipocytes and enhancing MMP-9 expression in tumor tissues, eventually favoring tumor angiogenesis [39]. Our study points out that NLRC4 expression in PDAC tissues was significantly higher than that in normal tissues, and patients with higher expression levels had worse prognoses, which may be connected to the tumor-promoting effect of NLRC4 mentioned above. Furthermore, BA significantly inhibited NLRC4 expression in Mia PaCa-2 cells, suggesting that it may be the target of BA’s anticancer and anti-inflammatory mechanisms.

## 4. Materials and Methods

### 4.1. Cell Culture

Human PDAC cell lines (Mia PaCa-2 and SUIT-2) and a nonmalignant pancreatic epithelial (hTERT-HPNE) cell line were obtained from Drs. Wun-Shaing Wayne Chang and Li-Tzong Chen, National Institute of Cancer Research (National Health Research Institutes, Miaoli, Taiwan). Mia PaCa-2 cells were cultured in high-glucose Dulbecco’s modified Eagle’s medium (DMEM) (Thermo Fisher Scientific, Waltham, MA, USA) supplemented with 10% fetal bovine serum (FBS; Corning), 2.5% horse serum, and 1% penicillin-streptomycin (TOKU-E, Bellingham, WA, USA). SUIT-2 cells were cultured in RPMI-1640 medium supplemented with 10% FBS and 1% penicillin-streptomycin. hTERT-HPNE cells were cultured in low-glucose DMEM (Hyclone, Thermo Scientific) supplemented with 5% FBS, 1% penicillin-streptomycin, and 10 ng/mL epidermal growth factor (Sino Biological, Wayne, PA, USA). These cells were free of mycoplasma contamination, and their identities were confirmed by short tandem repeat (STR) profiling at the BCRC and Center for Genomic Medicine, National Cheng Kung University (NCKU; Tainan, Taiwan). All cells were maintained at 37 °C in a 5% CO_2_ atmosphere. For drug treatment, cells were pre-cultured to 60–70% confluence and supplemented with medium containing BA (Sigma-Aldrich, St. Louis, MO, USA) for 24 or 48 h at indicated doses.

### 4.2. Betulinic Acid (BA)

BA (catalog no. 472-15-1) used in this study was purchased from Sigma Aldrich (St. Louis, MO, USA) with a purity of ≥98% (high-performance liquid chromatographic grade). A 10 mM stock solution was then prepared using dimethyl sulfoxide (DMSO) as a solvent and stored at −20 °C for subsequent experiments.

### 4.3. Cytotoxicity Assay

PDAC cells were seeded in 96-well plates at 5000 cells/well and cultured for 24 h before drug treatment. Fresh medium at 100 μL/well containing 0.2–0.5 μM BA was added to 96-well plates for 24 and 48 h. Thereafter, 50 μL of 1-(4,5-dimethylthiazol-2-yl)-3,5-diphenyl- formazan (MTT) (5 mg/mL) (Sigma-Aldrich, St. Louis, MO, USA) was added to each well of the 96-well plates and incubated for 4 h. Medium was replaced with 100 μL/well of DMSO. The absorbance was measured at 570 nm using an EPOCH2 microPlate Spectrophotometer (BioTek, Winooski, VT, USA), and the background absorbance at 630 nm was deducted.

### 4.4. Wound-Healing Assay

Mia PaCa-2 and SUIT-2 cells were seeded in 12-well plates. Once cells were 95–100% confluent, the growth medium was replaced with serum-free medium and incubated at 37 °C for 3 h in order to starve the cells. A horizontal scratch was made in the middle of each well using a sterilized 200 μL pipette tip. Growth medium with 0, 0.5, and 1 μM BA was added and then incubated for 24 and 48 h. Cell migration was immediately examined and recorded using phase-contrast microscopy, and at 24 and 48 h after scratching. Gap areas were quantified with ImageJ software to calculate the cell migratory ability based on the following formula: Cell migratory ability = (initial scratched area − post-treatment scratched area) ÷ initial scratched area.

### 4.5. Proteomics Analysis

Based on the previously obtained 50% inhibitory concentration (IC50) value of BA against Mia PaCa-2 cells, BA at concentrations of 0, 0.5, and 1 μM, which did not affect cell survival, were added to Mia PaCa-2 cells for a 24 h incubation. Cell pellets were then collected at 24 h for a subsequent mass spectrometry-based proteomics analysis. In brief, cells were lysed by ultrasonic vibration on ice for 2 min, and a bicinchoninic acid (BCA) assay was performed for protein quantification. Next, dithiothreitol (DTT) and iodoacetamide (IAA) were respectively added to reduce disulfide bonds and prevent them from re-annealing. Proteins were digested into peptide fragments with lysine protease and trypsin. After quantification, the same number of peptides from each sample were fractionated by basic reverse-phase fractionation [40] for tandem mass tag (TMT) labeling and desalting. Subsequently, peptide identification and quantitative analysis were implemented using the Thermo orbitrap fusion tribrid mass spectrometer (MS) with subsequent MaxQuant [41] and Perseus [42] analytical software. A one-way analysis of variance (ANOVA) was used to perform statistical tests to compare expression levels of all detected proteins under BA treatment at concentrations of 0, 0.5, and 1 μM. Moreover, differentially expressed proteins (DEPs) with similar patterns of change were classified into the same clusters, and the top five up- and downregulated genes were screened by a significant analysis of microarray (SAM) analysis for further data mining.

### 4.6. Bioinformatics Analysis

In light of the microarray data by the proteomics analysis, protein-coding genes of BA-induced upregulated (cluster 4), downregulated (cluster 6), and dose-dependent downregulated (cluster 7) proteins were imported into the Database for Annotation, Visualization and Integrated Discovery (DAVID) (https://david.ncifcrf.gov/tools.jsp, accessed on 26 November 2019) for a gene set enrichment analysis (GSEA). There were two annotation categories included in the current study, namely the Kyoto Encyclopedia of Genes and Genomes (KEGG) to search for biochemical reaction pathways involved in the genome, and the Gene Ontology (GO) database which contains three gene functional annotation terms: cellular components (CCs), molecular functions (MFs), and biological processes (BPs). In addition, the most significant up- and downregulated DEPs determined by the SAM analysis were further inputted into the Oncomine database (http://www.oncomine.org, accessed on 26 November 2019) to compare expressions of target protein-coding genes between clinical PDAC and normal pancreatic tissue specimens in existing cancer microarray datasets, and into the Kaplan–Meier (KM) plotter database (http://kmplot.com/analysis/, accessed on 26 November 2019) to observe whether PDAC patient survival was correlated with target DEP expression patterns.

### 4.7. Statistical Analysis

All cell experiments were performed in triplicate, and results are expressed as the mean ± standard error of the mean (SEM). All statistical analyses were performed with GraphPad Prism 6.0 software, and a one-way ANOVA with Tukey’s post-hoc test was used to compare differences among multiple groups. A false discovery rate (FDR) of <0.01 was defined in the SAM analysis of the protein microarray data. A *p*-value of < 0.001 was set as the threshold in the GO and KEGG annotation, and <0.05 as the threshold in the Oncomine and KM plotter database analyses.

## 5. Conclusions

Our study showed that BA can inhibit the survival rate of PDAC cells and reduce the migratory ability of PDAC cells. From a proteomics view, we assumed that these effects are possibly due to alterations of mitochondrial functions by BA, including decreases in the mitochondria complex I activity and oxidative phosphorylation, and downregulation of POLRMT and TACO1 expressions. In addition, BA-induced upregulation of APOA1 and downregulation of NLRC4 may provide a new explanation for the anti-inflammatory and antimetastatic effects of BA. However, whether these DEPs affected by BA can be implemented as the main target of PDAC therapy requires further in vivo and in vitro experiments for verification.

## Figures and Tables

**Table 1:** The 50% inhibitory concentration (IC_50_) values of pancreatic ductal adenocarcinoma (PDAC) cell lines (Mia PaCa-2 and SUIT-2) and normal pancreas cell lines (hTERT-HPNE) under betulinic acid (BA) treatment for 24 and 48 h.

	Cell Lines	Mia PaCa-2	SUIT-2	hTERT-HPNE
IC_50_				
**24 h**		1.783 μM	0.505 μM	5.689 μM
**48 h**		2.908 μM	0.864 μM	7.601 μM

IC_50_ values were calculated by Graphpad Prism 6.0, using the equation of “sigmoidal, 4PL” as a non-linear regression model.

## Data Availability

The data presented in this study are available on request from the corresponding author. The data are not publicly available due to privacy.

## Supplementary Materials

The following are available online at, Figure S1: Effects of betulinic acid (BA) on pancreatic ductal adenocarcinoma (PDAC) and normal cell viability, Table S1: Gene list of betulinic acid (BA)-induced differentially expressed proteins (DEPs) in cluster 4, Table S2: Gene list of betulinic acid (BA)-induced differentially expressed proteins (DEPs) in cluster 6, Table S3: Gene list of betulinic acid (BA)-induced differentially expressed proteins (DEPs) in cluster 7, Table S4: GO and KEGG analysis of differentially expressed proteins (DEPs) in cluster 4 (upregulation), Table S5: GO and KEGG analysis of differentially expressed proteins (DEPs) in cluster 6 (downregulation), Table S6: GO and KEGG analysis of differentially expressed proteins (DEPs) in cluster 7 (dose-dependent downregulation), Table S7: KM plot analysis of upregulated proteins induced by betulinic acid (BA), Table S8: KM plot analysis of downregulated proteins induced by betulinic acid (BA).

## Sample Availability

The samples presented in this study are available on request from the corresponding author.
