# Supplementary material for: Betulinic Acid Affects the Energy-Related Proteomic Profiling in Pancreatic Ductal Adenocarcinoma Cells
Source: Molecules. 2021 Apr 24;26(9):2482. doi: 10.3390/molecules26092482 (PMC8123215; doi:10.3390/molecules26092482)
Supplement: Supplementary file 1 [file molecules-26-02482-s001.zip › molecules-1192854-supplementary.pdf]

Supplementary Materials

Betulinic Acid Affects the Energy-Related Proteomic Profiling In Pancreatic Ductal Adenocarcinoma Cells

Ching-Feng Chiu [1,2,3], Hsin-Yi Chang [1,4,5], Chun-Yine Huang [6], Chen-Zou Mau [1], Tzu-Ting Kuo [7], Hsiu-Chuan Lee [6] and Shih-Yi Huang [1,2,6]

1 Graduate Institute of Metabolism and Obesity Sciences, Taipei Medical University, Taipei 11031, Taiwan
2 Nutrition Research Center, Taipei Medical University Hospital, Taipei 11031, Taiwan
3 TMU Research Center of Cancer Translational Medicine, Taipei Medical University, Taipei 11031, Taiwan
4 Graduate Institute of Cancer Biology and Drug Discovery, College of Medical Science and Technology, Taipei Medical University, Taipei 11031, Taiwan
5 Master Program in Clinical Pharmacogenomics and Pharmacoproteomics, College of Pharmacy, Taipei Medical University, Taipei 11031, Taiwan
6 School of Nutrition and Health Sciences, Taipei Medical University, Taipei 11031, Taiwan
7 Ph.D. Program for Cancer Molecular Biology and Drug Discovery, College of Medical Science and Technology, Taipei Medical University and Academia Sinica, Taipei 11031, Taiwan

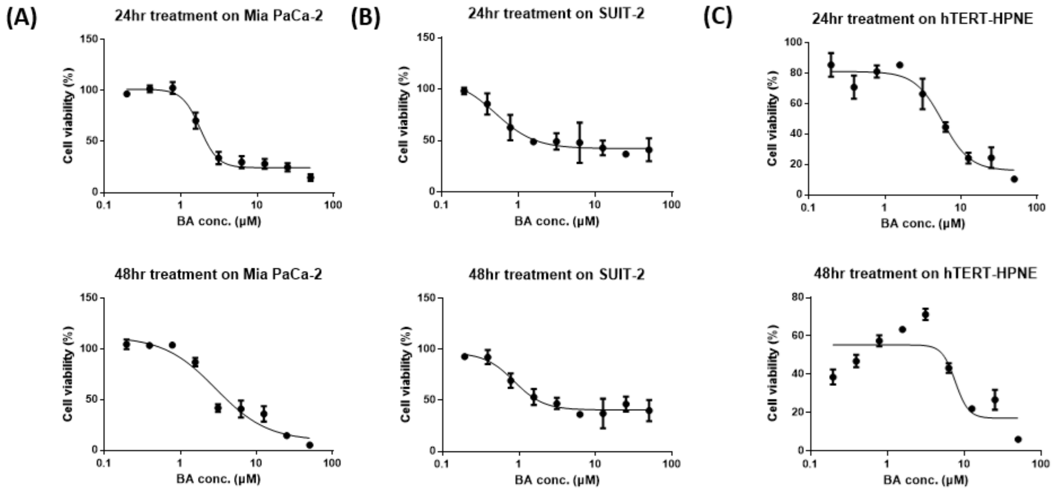

Figure S1. Effects of betulinic acid (BA) on pancreatic ductal adenocarcinoma (PDAC) and normal cell viability. Cells were treated with various concentrations of BA up to the maximal concentration of 50 μM for 24 and 48 h. (**A, B**) The 50% inhibitory concentration (IC50) values of Mia PaCa-2 and SUIT-2 cells indicated the cell viability of PDAC cell lines. (**C**) The IC50 value of hTERT-HPNE cells indicated the cell viability of normal pancreatic ductal cells. All experiments were performed in triplicate, and data are expressed as the mean ± SEM (n = 3).

Table S1. Gene list of betulinic acid (BA)-induced differentially expressed proteins (DEPs) in cluster 4.

Differential expression	Gene symbol of BA-regulated proteins
Upregulation	GOLIM4, EXOC5, NDUFA4, SDCBP, TOR1A, STX16, ZFP42, P4HA2, YKT6, HNRNPR, ASNA1, NARS, NUDT21, MED14, SYNCRIP, HIST1H2BL, PTCD1, NDUFS2, ZPR1, VPS4B, FLNB, UTP20, ATP5H, FLOT1, CIAO1, FSBP, VAPB, IPO7, MOCS3, PCNT, PTBP3, SDPR, CLPTM1, SOD1, PLAU, HLA-DRA, TFRC, TK1, RPN1, SLC4A2, SLC25A5, RPLP1, CDK1, CAPN1, APRT, LAMB1, HMBS, IGFBP1, DLD, IFIT1, UMPS, MTHFD1, PRKAR2A, PRKCSH, NME1, CD44, UBTF, PSMC3, RPL7, SDHB, FBL, AHCY, EEF1B2, ITGA3, EEF1G,

	DPP4, APEX1, CANX, HLA-DMA, HLA-DMB, PSMB6, EPHA2, PRDX3, PPIF, HMOX2, ALDH1B1, ATIC, STIP1, MCM7, SHMT1, RFC2, EIF4A3, DDOST, MDH1, RBM34, ECE1, RANBP1, RPS10, GYG1, RPL29, GSS, RPL34, NASP, PCYT1A, PPM1F, TMED10, GNAQ, ST13, RPL14, ANXA11, DAP3, HCFC1, HDGF, RAP1GDS1, NUP98, BLVRA, NUBP1, ALDH18A1, NAPA, ADK, SUMO4, BCAR1, RPS20, S100A10, RAB5B, RAB10, UBE2K, PSMC1, RPS23, RPS18, RPS29, RPS11, RPS25, RPS28, GNB2, RPS27A, ACTA1, GTF2I, MRPS22, MRPS11, RHOG, FKBP3, GBE1, SSBP1, EXOSC9, BCL2L1, SRSF1, PCDH1, DHX9, NUP160, DLG1, PTP4A2, PRDX4, SRSF6, G3BP1, PABPC4, TCOF1, NAE1, MTMR2, BLMH, ZMYM3, TRIM25, FAM50A, MESDC2, EIF4H, WDR43, RNPS1, RBBP7, TIMM50, PARP14, TBC1D10B, CYBRD1, ATAD3B, LAMTOR1, DHRS7B, C8orf82, DHRS4L2, MPRIP, BTN2A1, DHX30, EIF3M, DCXR, AGPAT6, WDR75, ALDH16A1, EIF1AD, SPC24, UXS1, LEMD2, FAM98A, SCARB1, PDCD6IP, UBXN4, LARP4B, PRCC, INTS12, MRRF, CYFIP2, LTV1, COQ2, HPDL, THOC3, CNDP2, MIA2, VPS35, ERGIC2, PIGS, SRPK1, PSMB7, EIF3C, COPS8, SH3GL1, DPH2, AGMAT, GNL3, RTFDC1, GTPBP4, PITHD1, TMEM126A, DHX36, TRABD, ACAD9, ECT2, XAB2, PREB, MRPL47, NANS, MBNL1, OSTC, VPS45, PNO1, OLA1, DDX18, SLTM, THUMPD1, BRE, TERF2IP, MAT2B, COMMD9, THYN1, TOMM7, BCCIP, ORC3, EIF3K, GRHPR, CLIP2, NIPSNAP3A, RSL24D1, SFMBT1, ENOPH1, VPS51, BAG5, DNPEP, MAGED2, SRRM2, POMP, GINS2, PLAA, RCL1, SH3GLB1, MRPS23, RPL36, PRKAB1, TBL2, LAS1L, HSPB11, PPME1, SUPT16H, ZNF706, ALG5

Table S2. Gene list of betulinic acid (BA)-induced differentially expressed proteins (DEPs) in cluster 6.

Differential expression	Gene symbol of BA-regulated proteins
Downregulation	ILVBL, FAM83G, ATOX1, MEN1, PIR, SCD, DYNC1LI2, NDUFB5, NDUFB3, DHX16, UGDH, PLXNA2, NDUFS3, ECI2, IDH1, DHRS3, USP19, SBF1, NDUFA10, ETHE1, OAS1, OAT, PRKCB, LGALS2, S100A6, EPHX1, GPX1, CTSB, RHOC, RBP1, COX4I1, VCAN, ETFA, COX7A2, AKR1B1, GM2A, HK1, M6PR, CYP3A5, BCKDHB, TGM2, SP100, FKBP2, MAP4, NDUFS1, PML, BLVRB, ATP5D, PEBP1, PPP2R1B, AXL, S100A11, ADD1, SRP14, CAPG, EIF1, AKR1C3, ME1, ACADVL, NDUFV1, EMD, HSD17B4, AKR1D1, SUCLG1, NDUFA6, TMEM33, DAD1, PPP1CA, TMSB10, NUCB2, MRPS21, ARF5, BDH1, SSRP1, GALNT1, PRKAA1, PUM2, MVP, PSMD6, NDUFA5, PCK2, DPY19L1, PHLDA2, HSD17B12, ACBD5, INTS3, WDR74, VPS13C, HDGFRP2, PPFIBP1, NDUFA11, AHNAK2, FUNDC1, FAM114A1, PARP9, TRMT2A, RDH10, INTS1, MCFD2, DTX3L, ERGIC1, SYAP1, PTCD3, S100A16, PRRC1, C8orf37, XPO6, MCCC1, CLCC1, CDC5L, TXN2, ACO2, TACO1, TBCD, MRPL37, INTS2, RNF167, FN3KRP, UGT1A8, ACOT13, NLRC4, MRPL40, POLE3, UQCC1, TMEM38B, RBM28, PPP1R1B, AASS, NDUFA12, PYCARD, VDAC3, MRPS7, OAS3, SLC4A7, DDX49

In total, 135 downregulated proteins in cluster 6 were identified by a proteomics analysis after 0.5 and 1 μM BA treatment for 24 h in Mia PaCa-2 cells.

Table S3. Gene list of betulinic acid (BA)-induced differentially expressed proteins (DEPs) in cluster 7.

Differential expression	Gene symbol of BA-regulated proteins
Dose-dependent downregulation	SNRPG, PDLIM1, POLRMT, LAD1, CASK, HSPA12A, DENR, XPOT, TBC1D4, MGEA5, DIAPH1, SH3BGRL, SRSF10, DDAH2, ADH1C, AK1, CA2, ALDOA, HIST1H2AJ, ALDH2, PTMA, ENO1, TPM3, HIST2H2BE, DBI, LDHB, HSP90AA1, HSP90AB1, GSTP1, FBP1, LTA4H, HSPA1B, ESD, PYGB, ADH5, PCNA, AKR1A1, PKM, DSP, HIST1H1B, HIST1H1C, STMN1, PFKL, LGALS3, VCL, PGAM1, PTMS, GSTM3, ACO1, UGT1A1, CFL1, DTYMK, MCM3, S100A4, HMGB2, PSMB4, LAP3, MARCKS, GCHFR, CMPK1, SRI, SERPINB1, HIBADH, HNRNPH3, PRDX2, ABCD1, GLRX, CHKA, ARR3, SERPINB5, HSD17B2, TALDO1, RPL35, ALDH1A3, ARRB1, AARS, SARS, SUOX, HMGCS2, MARS, LGALS4, ACTB, HNRNPK, HIST1H4A, RAN, PPIA, FKBP1A, YWHAZ, EIF5A, ACTG1, TUBA1B, ARF1, CYCS, SORD, AMPD2, ACY1, PSME1, PRDX1, ECH1, COASY, MTAP, CBX3, SELENBP1, PDAP1, FKBP5, TTLL12, PLS1, PTGR1, PPA1, MAPRE1, ADIRF, DDB1, ACSF3, ACTBL2, SGOL1, TTC38, TWF2, LPCAT3, APOA5, CRELD2, EFTUD1, CSMD3, SCCPDH, PIP4K2C, INTS4, PHACTR3, CMSS1, TUBA1C, FERMT1, ASPSCR1, DPY30, NT5C3A, UGT1A10, XPNPEP1, PHPT1, RBM22, CDKN2AIP, ATXN10, COPG2, STK39, LIMA1, MYO6, CARHSP1

In total, 141 dose-dependent downregulated proteins in cluster 7 were identified by a proteomics analysis after 0.5 and 1 μM BA treatment for 24 h in Mia PaCa-2 cells.

Table S4. GO and KEGG analysis of differentially expressed proteins (DEPs) in cluster 4 (upregulation).

GO category	Gene function	Count	%	*p* value
BP	rRNA processing	25	9.7	1.10×10^{-14}
BP	translational initiation	20	7.8	1.50×10^{-13}
BP	viral transcription	17	6.6	8.50×10^{-12}
BP	nuclear-transcribed mRNA catabolic process, nonsense-mediated decay	17	6.6	2.20×10^{-11}
BP	SRP-dependent cotranslational protein targeting to membrane	15	5.8	9.90×10^{-11}
BP	cell-cell adhesion	21	8.2	3.90×10^{-9}
BP	translation	20	7.8	7.20×10^{-9}
BP	RNA export from nucleus	7	2.7	1.50×10^{-4}
BP	ribosome biogenesis	6	2.3	1.80×10^{-4}
BP	cytoplasmic translation	5	1.9	4.60×10^{-4}
CC	extracellular exosome	100	38.9	2.30×10^{-20}
CC	cytosol	103	40.1	7.60×10^{-17}
CC	membrane	77	30	1.40×10^{-14}
CC	ribosome	21	8.2	1.70×10^{-13}
CC	nucleoplasm	81	31.5	4.00×10^{-11}
CC	cell-cell adherens junction	23	8.9	9.20×10^{-10}
CC	focal adhesion	25	9.7	1.20×10^{-9}
CC	small ribosomal subunit	8	3.1	6.40×10^{-8}
CC	cytoplasm	113	44	7.50×10^{-8}
CC	nucleolus	34	13.2	1.10×10^{-7}
CC	cytosolic small ribosomal subunit	9	3.5	2.90×10^{-7}
CC	mitochondrion	43	16.7	4.40×10^{-7}
CC	melanosome	10	3.9	1.10×10^{-5}
CC	myelin sheath	11	4.3	5.30×10^{-5}
CC	cytosolic large ribosomal subunit	7	2.7	3.60×10^{-4}
CC	mitochondrial inner membrane	17	6.6	4.60×10^{-4}
MF	poly(A) RNA binding	69	26.8	1.50×10^{-24}
MF	protein binding	181	70.4	2.20×10^{-11}
MF	structural constituent of ribosome	21	8.2	1.10×10^{-10}
MF	cadherin binding involved in cell-cell adhesion	22	8.6	2.00×10^{-9}
MF	RNA binding	28	10.9	3.90×10^{-8}

KEGG	Pathway	Count	%	*p* value
	Ribosome	17	6.6	1.80×10^{-8}

p values of < 0.001 were included in the following list. GO, gene ontology; KEGG, Kyoto Encyclopedia of Genes and Genomes; BP, biological process; CC, cellular component; MF, molecular function.

Table S5. GO and KEGG analysis of differentially expressed proteins (DEPs) in cluster 6 (down-regulation).

GO category	Gene function	Count	%	*p* value
BP	mitochondrial electron transport, NADH to ubiquinone	10	7.4	9.70×10^{-11}
BP	mitochondrial respiratory chain complex I assembly	10	7.4	1.00×10^{-9}
BP	oxidation-reduction process	17	12.6	1.00×10^{-5}
CC	mitochondrion	38	28.1	4.80×10^{-13}
CC	mitochondrial respiratory chain complex I	10	7.4	6.70×10^{-11}
CC	mitochondrial matrix	17	12.6	1.90×10^{-9}
CC	mitochondrial inner membrane	19	14.1	3.10×10^{-9}
CC	extracellular exosome	39	28.9	6.60×10^{-5}
MF	NADH dehydrogenase (ubiquinone) activity	9	6.7	2.90×10^{-9}
MF	electron carrier activity	9	6.7	4.50×10^{-7}
MF	oxidoreductase activity	9	6.7	1.60×10^{-4}
KEGG	Pathway	Count	%	*p* value
	Metabolic pathways	37	27.4	1.40×10^{-9}
	Parkinson's disease	14	10.4	3.00×10^{-9}
	Huntington's disease	15	11.1	1.40×10^{-8}
	Oxidative phosphorylation	13	9.6	1.50×10^{-8}
	Non-alcoholic fatty liver disease (NAFLD)	13	9.6	6.40×10^{-8}
	Alzheimer's disease	13	9.6	2.10×10^{-7}

p values of < 0.001 were included in the following list. GO, gene ontology; KEGG, Kyoto Encyclopedia of Genes and Genomes; BP, biological process; CC, cellular component; MF, molecular function.

Table S6. GO and KEGG analysis of differentially expressed proteins (DEPs) in cluster 7 (dose-dependent downregulation).

GO category	Gene function	Count	%	*p* value
BP	cell-cell adhesion	14	10	2.40×10^{-7}
BP	canonical glycolysis	5	3.6	4.90×10^{-5}
CC	extracellular exosome	84	60	1.20×10^{-33}
CC	cytosol	74	52.9	1.40×10^{-20}
CC	cytoplasm	75	53.6	3.80×10^{-10}
CC	cell-cell adherens junction	16	11.4	2.10×10^{-8}
CC	focal adhesion	15	10.7	1.50×10^{-6}
CC	myelin sheath	9	6.4	2.00×10^{-5}
CC	extracellular space	26	18.6	2.20×10^{-5}
CC	membrane	35	25	2.80×10^{-5}
CC	mitochondrion	25	17.9	5.20×10^{-5}
CC	extracellular matrix	11	7.9	8.20×10^{-5}
MF	poly(A) RNA binding	31	22.1	4.00×10^{-9}
MF	cadherin binding involved in cell-cell adhesion	16	11.4	1.10×10^{-8}
MF	identical protein binding	22	15.7	5.10×10^{-7}
MF	oxidoreductase activity	11	7.9	4.70×10^{-6}
MF	ion channel binding	7	5	2.90×10^{-4}
KEGG	Pathway	Count	%	*p* value
	Glycolysis/Gluconeogenesis	12	8.6	4.40×10^{-10}
	Biosynthesis of antibiotics	16	11.4	4.10×10^{-8}
	Carbon metabolism	10	7.1	1.00×10^{-5}
	Biosynthesis of amino acids	8	5.7	2.80×10^{-5}
	Metabolic pathways	32	22.9	3.60×10^{-5}
	Drug metabolism - cytochrome P450	7	5	1.90×10^{-4}
	Metabolism of xenobiotics by cytochrome P450	7	5	3.00×10^{-4}
	Chemical carcinogenesis	7	5	4.60×10^{-4}

p values of < 0.001 were included in the following list. GO, gene ontology; KEGG, Kyoto Encyclopedia of Genes and Genomes; BP, biological process; CC, cellular component; MF, molecular function.

Table S7. KM plot analysis of upregulated proteins induced by betulinic acid (BA).

Gene name	Survival type	Number at risk		Median survival		*p* value
		low	high	Low-expression cohort (months)	High-expression cohort (months)	
**OSTC**	**OS**	81	96	35.3	17.27	0.002*
	RFS	33	36	42.9	9.7	0.0003*
**RPS18**	OS	71	106	23.03	17.23	0.0046*
	RFS	49	20	NA	NA	0.0219*
**VAPB**	OS	114	63	19.77	24.4	0.0311*
	RFS	17	52	23.87	16.2	0.331
**NDUFS2**	OS	47	130	67.87	18.93	0.0316*
	RFS	19	50	50.37	27.7	0.0303*
**APOA1**	OS	58	119	15.87	23.17	0.0018*
	RFS	48	21	16.4	27.7	0.1184
**BRE (BABAM2)**	OS	111	66	19.93	20.23	0.2511
	RFS	48	21	12.6	42.9	0.0109*

*$p < 0.05$ was considered to show a statistically significant difference between the high- and low-expression cohorts. OS, overall survival; RFS, recurrence-free survival.

Table S8. KM plot analysis of downregulated proteins induced by betulinic acid (BA)

Gene name	Survival type	Number at risk		median survival		*p* value
		low	high	Low-expression cohort (months)	High-expression cohort (months)	
**NLRC4**	**OS**	66	111	24.4	19.77	0.0745
	RFS	20	49	50.37	16.2	0.0033*
**INTS2**	OS	52	125	18.93	20.23	0.3832
	RFS	21	48	50.37	16.4	0.1719
**DDX49**	OS	44	133	18.17	22.8	0.0108*
	RFS	47	22	16.4	32.9	0.1804
**TACO1**	OS	132	45	19.77	67.87	0.0012*
	RFS	43	26	14.97	18.07	0.0697
**RNF167**	OS	64	113	15.27	23.17	0.0000032*
	RFS	17	52	12.6	50.37	0.0003*
**POLRMT**	OS	52	125	16.2	23.03	0.0108*
	RFS	47	22	NA	NA	0.0075*
**ADH1C**	OS	122	55	21.13	18.17	0.0938
	RFS	40	29	50.37	20.67	0.2142

*$p < 0.05$ was considered to show a statistically significant difference between the high- and low-expression cohorts. OS, overall survival; RFS, recurrence-free survival.
